# Idiopathic Bullous Pemphigoid With Concurrent Gingival and Cutaneous Involvement: A Case Report

**DOI:** 10.7759/cureus.89933

**Published:** 2025-08-12

**Authors:** Angela R Loczi-Storm, Alyssa M Iurillo, Fatima N Mirza, Paige Clement, Oliver J Wisco

**Affiliations:** 1 College of Osteopathic Medicine - Northwest, Western University of Health Sciences, Lebanon, USA; 2 Dermatology, Indiana University School of Medicine, Indianapolis, USA; 3 Dermatology, The Warren Alpert Medical School of Brown University, Providence, USA; 4 Dermatology, University of Sterling, Bend, USA

**Keywords:** autoimmune bullous, bullous pemphigoid (bp), drug induced bullous pemphigoid, immune mucosal, mucosal

## Abstract

Bullous pemphigoid (BP) is the most common autoimmune blistering disease, primarily affecting older adults and typically presenting with intensely pruritic, tense cutaneous bullae. While mucosal involvement is more common in other blistering disorders such as mucous membrane pemphigoid, it can occasionally occur in BP. This atypical presentation may confound diagnosis and cause treatment delays. Here, we report the case of a 67-year-old woman with idiopathic BP involving both cutaneous and oral mucosal surfaces. The disease began with a single bulla on the ear and a pruritic rash across the upper back, eventually progressing to widespread cutaneous bullae with painful gingival involvement. After a suboptimal response to prednisone and mycophenolate mofetil, remission was successfully achieved with rituximab. This case underscores the diagnostic complexity and treatment challenges often seen in BP with mucosal involvement. Early recognition of atypical variants and careful histopathologic assessment are essential for guiding effective therapy.

## Introduction

Bullous pemphigoid (BP) is the most common subepidermal blistering disorder that predominantly affects geriatric populations [1,2]. In the United States, the annual incidence of BP is between 2.4 and 23 cases per million in the general population, but in individuals over the age of 70, the number of annual cases is as high as 190-312 per million [3]. BP can present in several clinical variants defined by age of onset, distribution of lesions, and potential triggers [1,2]. This heterogeneity contributes to diagnostic challenges, as presentations can range from classic tense bullae on erythematous skin to atypical forms involving urticarial, eczematous, or even mucosal lesions [1].

## Case presentation

A 67-year-old woman presented with a two-year history of blistering affecting the skin and oral mucosa. Her symptoms began with a bulla on the left ear, along with a severely pruritic, erythematous rash across the upper back. Her past medical history was unremarkable, although her mother had a history of sarcoidosis.

**Figure 1:**
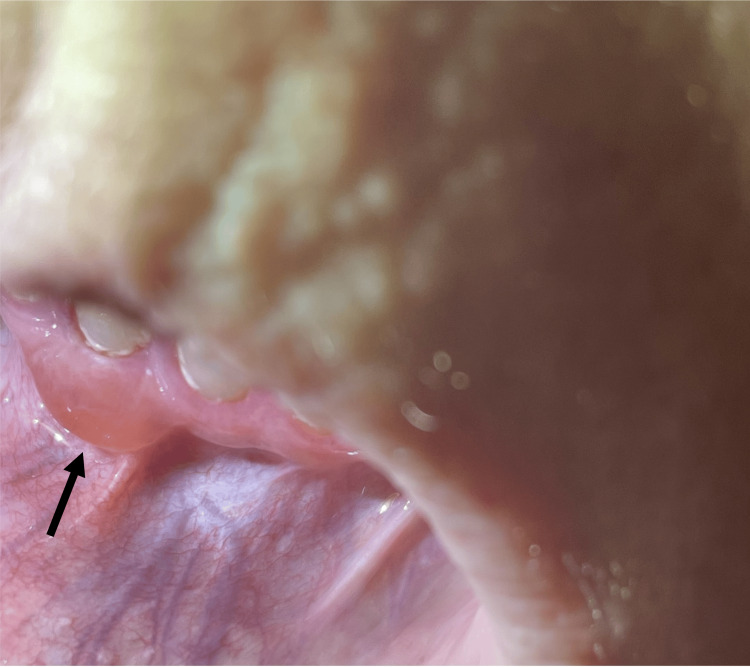
Tense, well-defined bulla on the lower gingival mucosa with mild surrounding erythema, consistent with a subepithelial blister.

Since the initial presentation, bullae predominantly manifested on the back, with additional occurrences noted on the upper and lower extremities, chest, scalp, abdomen, and oral mucosa. A single pharyngeal bulla was noted by an otolaryngologist. Oral involvement was characterized by painful gingival vesicles and bullae, ulcers, and gingivitis (Figure 1).

**Figure 2:**
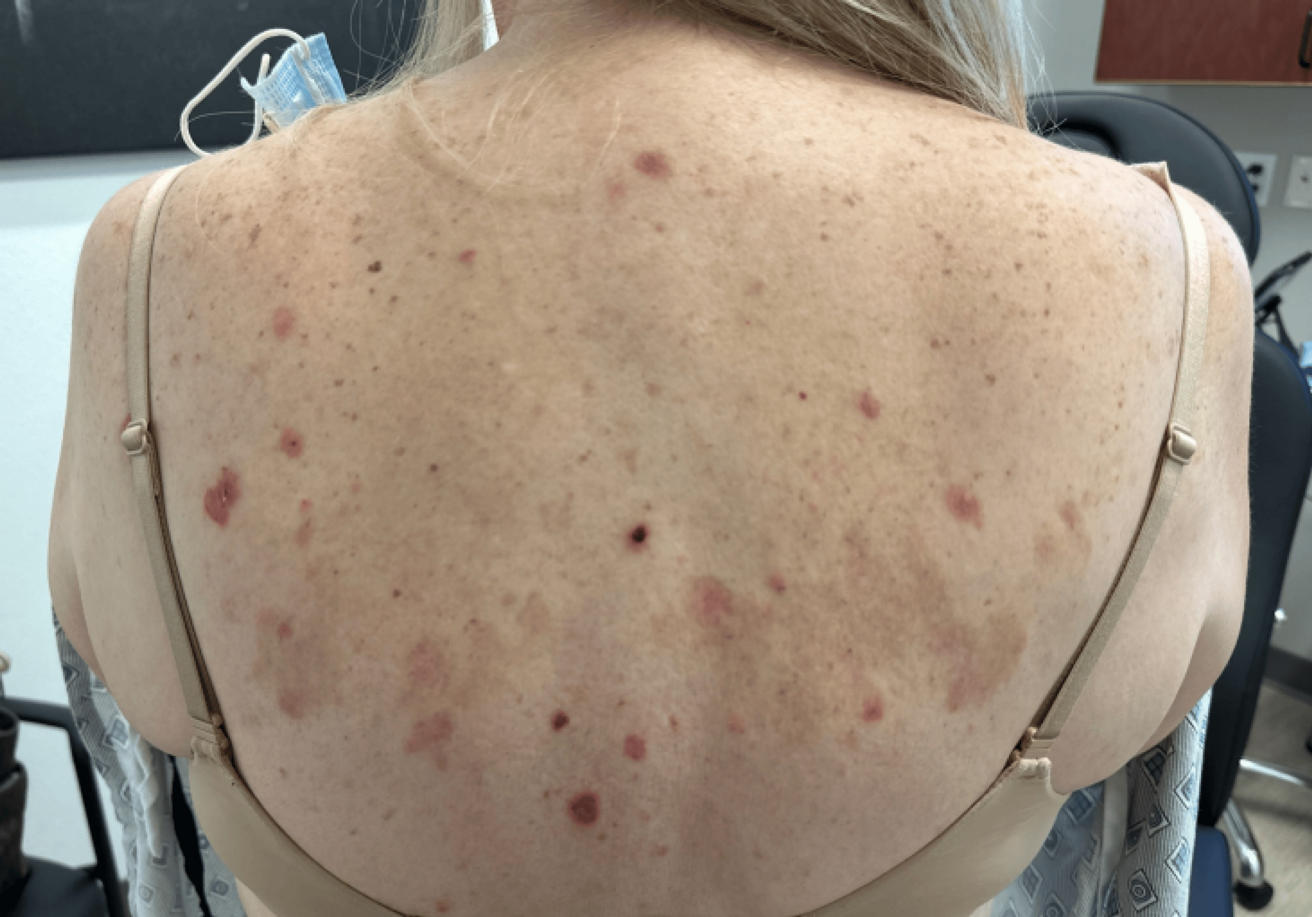
Scattered erythematous papules and plaques with central crusting and erosion on the upper back.

**Figure 3:**
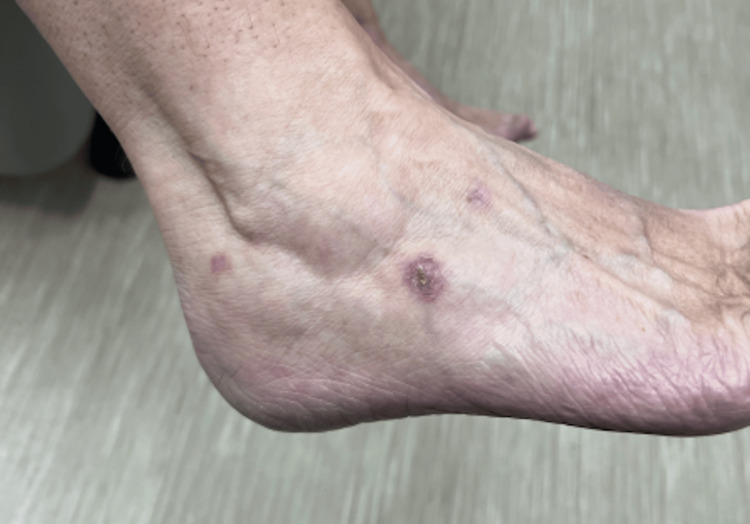
Erythematous plaque with central crusting and erosion on the lateral aspect of the right ankle.

Cutaneous outbreaks showed scattered indurated erythematous plaques, some with overlying firm bullae and others with overlying crust, preceded by significant pruritus (Figures 2, 3). 

The patient continued to develop new bullae across the back, chest, and gingiva, prompting a shave biopsy of the upper back by her previous dermatologist. Histologic examination revealed perivascular dermatitis with eosinophils, raising concern for an arthropod bite and resulting in a diagnosis of scabies. Her symptoms persisted despite completing two rounds of permethrin treatments.

A shave biopsy was reperformed, demonstrating subepidermal vesiculobullous dermatitis with eosinophils aligned along the basal layer. Direct immunofluorescence revealed linear deposition of IgG, IgG4, and C3 at the dermal-epidermal junction. Additionally, an ELISA detected elevated levels of IgG BP-180 antibodies, leading to a diagnosis of BP.

Upon presenting to our clinic, the patient was prescribed high-dose prednisone, doxycycline monohydrate 100 mg twice daily, and niacinamide 500 mg twice daily. For cutaneous and mucosal lesions, 0.5% clobetasol ointment twice daily and lidocaine solution before meals were prescribed as needed.

Doxycycline was later discontinued due to esophageal discomfort. Attempts to slowly taper prednisone below 20 mg daily resulted in blistering and pruritic flares. The patient was then started on mycophenolate mofetil 1000 mg twice daily, and prednisone was increased back to 20 mg daily. Over the following year, recurrent flares continued, and the patient was unable to successfully taper off prednisone.

Given the limited improvement, IV rituximab was initiated. At this time, the patient has been clear of disease since her second rituximab infusion and has since successfully tapered off prednisone. Recently, she has started tapering off mycophenolate mofetil.

## Discussion

BP is the most common autoimmune subepidermal blistering disorder, primarily affecting older adults [1]. It is part of a rare group of pemphigoid diseases characterized by autoantibodies targeting components of the dermal-epidermal adhesion complex [2,3,4]. Classic BP presents with tense bullae on erythematous skin, often accompanied by intense pruritus and urticarial plaques, with lesions typically affecting flexural surfaces of the limbs and lower abdomen [5]. Mucous membrane involvement, atrophic scarring, or predominant head and neck lesions are generally absent in typical BP [6].

Although mucosal involvement is uncommon, occurring in only 5-18% of cases, it can complicate diagnosis and delay treatment [4,5,7]. When present, mucosal lesions usually appear as erosions on non-keratinized sites such as the buccal mucosa or soft palate, as intact bullae tend to rupture easily [7]. Pharyngeal involvement is exceptionally rare, with only isolated case reports [7,8]. Most mucosal BP cases are limited to a single non-keratinized oral site, with multifocal oral involvement or concurrent pharyngeal lesions being exceedingly rare [7]. When mucosal lesions are present, they are typically accompanied by cutaneous eruptions concentrated on the head and neck [4]. In contrast, although our patient’s initial bulla appeared on the ear, the majority of their cutaneous disease involved the back and trunk, deviating from the typical distribution. Additionally, the predilection of bullae to form on the keratinized gingiva in this case is particularly unusual [6]. Mucosal involvement in BP is clinically important because it is often associated with more severe disease, a higher risk of treatment resistance, and prolonged healing times compared to purely cutaneous forms [4].

Variations in the immunopathogenesis of BP may contribute to such atypical presentations. BP is marked by the presence of circulating IgG autoantibodies targeting structural proteins within keratinocyte hemidesmosomes, most notably BP180 and BP230 [3,4]. In cases with mucosal involvement, autoantibodies have been found to primarily target the NC16A domain of BP180, and emerging evidence suggests that epitope heterogeneity may correlate with specific clinical phenotypes, including mucosal involvement with or without cutaneous disease [9].

External triggers and risk factors also influence disease patterns. Idiopathic BP in older adults typically manifests as isolated cutaneous disease, making mucosal involvement in this population particularly unusual [3,4]. Mucosal lesions are more frequently observed in drug-associated BP (DABP), renal allograft recipients, and younger patients [10,11]. Over 89 drugs have been implicated in DABP, with gliptins, PD-1/PD-L1 inhibitors, loop diuretics, and penicillin derivatives showing the strongest associations [10,12]. A thorough medication history is essential to identify potential offending agents and distinguish drug-induced disease from idiopathic BP. While clinical differentiation between idiopathic BP and DABP is challenging due to overlapping features, DABP often exhibits a rapid response to corticosteroid therapy, whereas idiopathic BP tends to follow a more protracted, treatment-resistant course as seen in our patient [12,13]. Rituximab, a monoclonal anti-CD20 antibody, has gained traction as an effective option for refractory BP [14]. In our patient, rituximab led to complete resolution of cutaneous and mucosal lesions after suboptimal response to corticosteroids and mycophenolate mofetil, highlighting its role as a valuable steroid-sparing therapy in resistant disease.

## Conclusions

This case underscores the importance of recognizing atypical presentations of BP, particularly when mucosal involvement is present without the expected head and neck distribution. The coexistence of gingival and pharyngeal lesions with predominantly trunk involvement highlights the need for heightened clinical vigilance, as such variants may challenge standard diagnostic and treatment approaches. By documenting these unusual patterns, we can improve awareness and refine clinical strategies for managing atypical presentations of BP.
